# Staff supported community outings among forensic mental health patients: patient characteristics, rehabilitative goals, and (the absence of) adverse outcomes

**DOI:** 10.3389/fpsyt.2024.1382676

**Published:** 2024-04-02

**Authors:** Christian Farrell, Karen L. Petersen, Peri Hanzouli, Tonia L. Nicholls

**Affiliations:** ^1^ Department of Psychiatry, University of British Columbia, Vancouver, BC, Canada; ^2^ Department of Psychiatry, University of Alberta, Edmonton, AB, Canada; ^3^ BC Mental Health and Substance Use Services, Provincial Health Services Authority, Coquitlam, BC, Canada; ^4^ Department of Psychology, Simon Fraser University, Burnaby, BC, Canada

**Keywords:** community access, forensic psychiatric patients, risk assessment, unauthorized leave, violence

## Abstract

Mental health professionals are tasked with making difficult clinical decisions in treatment settings. In the forensic system, decision making regarding staff supervised community outings (SSCOs) provides a significant challenge due to the need to balance patient liberties, mental health recovery, and public safety. This study explored the characteristics and rehabilitative nature of SSCOs, characteristics of patients attending SSCOs, and any adverse events that occurred during the outings. Employing a cross-sectional design, 110 patients who participated in SSCOs over a one-year period from a Canadian Forensic Psychiatric Hospital were included. Clinical records were reviewed to capture patient and SSCO variables. Descriptive analyses were used to calculate participant, risk, SSCO, and adverse event characteristics. Qualitative analysis was used to explore the purpose of SSCOs and rehabilitative progress that occurred during the outings. Patients attending SSCOs were comprised of long-stay patients with over half having committed a violent index offence. Almost 75% of patients had a moderate/high risk for violence and 50% of the patients had a moderate/high risk of absconding. During the study period, 463 SSCOs were completed. Most outings focused on developing skills for daily living and staff comments suggested many patients developed skills in these areas. Despite considerable risk profiles and public concern regarding forensic patients having community access, there was a single occurrence of unauthorized leave and no instances of violence or substance use. This research can disrupt stigma, demonstrating that SSCOs support a specific rehabilitative intent, promote community reintegration, and maintain public safety.

## Introduction

Mental health professionals are often required to make complex clinical decisions that frequently carry a high degree of uncertainty, given the lack of objective tools to support decision making (1). In forensic psychiatric settings, clinical decision making becomes particularly complex when it involves determining readiness for access to the community given this aspect of treatment is partly controlled by a tribunal; in Canada, this is termed a provincial Review Board. In these scenarios, the multidisciplinary treatment team is generally required to present estimations of risk of harm to the patient and the public to the Review Board, while also providing evidence to support the patient’s civil liberties and treating in the least restrictive environment.

In Canada, clinicians making these assessments and clinical decisions are informed by current Canadian legislation, primarily from the *Criminal Code of Canada* (CCC), which states that for individuals found Not Criminally Responsible on Account of Mental Disorder (NCRMD, the Canadian version of the insanity defense), dispositions should be based on “…the safety of the public, which is the paramount consideration, the mental condition of the accused, the reintegration of the accused into society and the other needs of the accused…” (2). This law demands a careful approach involving risk assessment and risk mitigation to ensure public safety is prioritized while supporting the NCRMD individual in mental health treatment, recovery, and community reintegration (3). It is, therefore, important for clinicians operating in forensic mental health services to make treatment decisions that can allow patients to safely participate in their communities and support their transition to community settings.

Many jurisdictions allow individuals in various secure settings (e.g., forensic mental health hospitals, substance use and psychiatric rehabilitation settings, prisons) to participate in various forms of community access, with the broad goals of fostering community relationships and building skills that will assist with eventual community re-integration (4). Community access has been found to be specifically relevant to the recovery of mentally ill individuals who have contact with the criminal justice system (4–6). In the forensic mental health system, Staff Supported Community Outings (SSCOs) promote the tenets of community reintegration and are also designed to support patients on their journey toward mental health recovery (7). Prioritizing community reintegration opportunities allows forensic patients to rebuild or maintain family ties, access community resources, and develop vocational and leisure skills (e.g., public transit skills, grocery shopping, and fitness). The importance of patients being able to practice their skills and generalize them to the social environment is a pillar of mental health recovery (8). Furthermore, the importance of community access is also consistent with leading theories guiding evidence-based practice with criminal justice involved individuals (e.g., the Risk, Needs, Responsivity model, RNR, (9); as well as the Good Lives Model, GLM; (10)). Lastly, community outings provide staff and decision makers essential information. Community access allows the care team to evaluate the patient in a safe, gradual manner as they experience and respond to an environment that is less controlled than a secure inpatient setting, which may reveal both strengths and vulnerabilities that are used to inform programming needs and future risk assessments.

There is growing interest internationally regarding community outings from forensic settings, including discussion around leave policies, staff and patient perceptions regarding the utility of short-term leaves, and the impact of leaves on treatment progress (11–13). Additionally, there is a robust body of research supporting the importance of vocational and leisure programing for criminal justice involved populations and specifically, forensic patients; however, authors of a systematic review concluded that more research is necessary (14). Despite these gains there has been minimal exploration into escorted leaves from secure forensic settings.

Given the need to support patient liberties and recovery, while prioritizing the safety of the public in the forensic system, robust research is needed to further understand the role supervised access to the community has in mental health recovery and risk management for forensic patients. Specifically, our study was designed to address three primary objectives (1): explore the sociodemographic, clinical, and risk characteristics of patients who attend SSCOs (2), examine the characteristics of SSCOs and their rehabilitative purpose, and (3) understand the frequency and nature of adverse events during the outings. These objectives could aid in our understanding of the potential therapeutic benefits of staff supported community access and help inform the clinical decision making involved in granting access to the community for forensic psychiatric patients.

## Methods

### Design and sample

We employed a cross-sectional design to capture all SSCOs that occurred in the only forensic psychiatric hospital in British Columbia, Canada, during a one-year period. Inclusion criteria required that patients attend at least one SSCO over the course of the study year (i.e., 2017). This typically included patients being treated under the following legal designations: Not Criminally Responsible on Account of Mental Disorder (NCRMD), Unfit to Stand Trial, or involuntary status under the BC Mental Health Act. Our final sample of participants who met inclusion criteria was 110. This study received ethics approval from the University of British Columbia as well as BC Mental Health and Substance Use Services.

### Setting

The BC forensic psychiatric services is a central system served by a single 190 bed hospital and seven clinics throughout the geographically diverse province. Patients are admitted to this facility under several circumstances, including the following (1): treatment under a Not Criminally Responsible on Account of Mental Disorder (NCRMD) or Unfit to Stand Trial finding, (2) assessment of NCRMD or Fitness to Stand Trial, and/or (3) transfer from jail for temporary treatment under the BC Mental Health Act. As patients who fall under the latter two categories (i.e., temporary treatment or assessment orders) typically have short stays (approximately 30 days, and generally are discharged back to the correctional center) and are admitted because they are acutely psychiatrically unwell, they generally do not participate in SSCOs.

**Figure 1:**
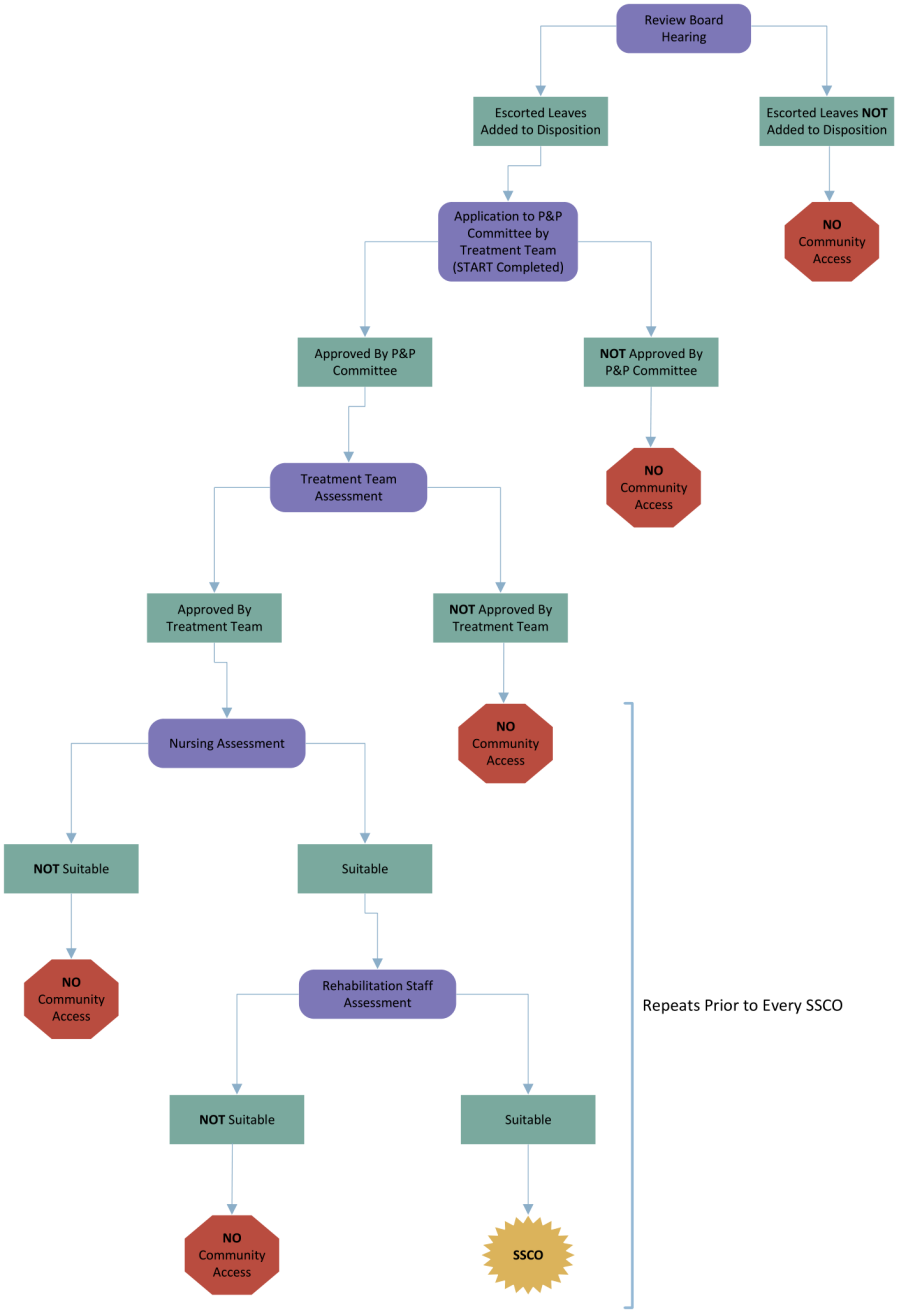
Process for forensic psychiatric patients to gain escort access to the community.

The hospital has a formal process in place, encompassing both a legal mandate and hospital policies, to ensure that SSCOs are safe for the patient and the community (see **Figure 1**). Section 672.38 (1) of the *Criminal Code of Canada* gives the provincial Review Board jurisdiction to make orders regarding detention, community access, leaves, and discharge. Before patients are considered for access to the community, the Review Board needs to add this order to the patient’s legal disposition. Once the Review Board has permitted access to the community, hospital policy requires that the treatment team apply to the Program & Privilege (P&P) committee, which is comprised of diverse professionals (e.g., nursing, social work, psychiatry, rehabilitation) and including a mix of senior hospital leadership and direct care clinicians. Only after the P&P committee grants SSCOs, does the patient’s interdisciplinary treatment team have the authority to permit community access. Importantly, the treatment team is responsible for continually assessing the patient’s mental state and, if concerns arise, can revoke community access at any time, including right up until the time when a person is scheduled for an outing. Finally, on the day of the planned leave into the community, a final assessment of the patient’s mental state is conducted by a member of the patient’s treatment team (e.g., nurse) in collaboration with the rehabilitation department staff member who is planning to accompany the patient on the outing. These staff members can decline to take a patient on an outing if they notice a deterioration in mental state or a change in the patient’s risk profile. Additionally, if during the outing staff have concerns about the patient’s behaviors or mental state, the SSCO can be terminated early, and the patient returned to the hospital.

### Procedure and data sources

Data was collected from patient clinical charts and hospital rehabilitation department records. Sociodemographic characteristics (e.g., age, gender, ethnicity, etc.), clinical variables (e.g., primary psychiatric diagnosis, prior contact with services, legal status, etc.), START summary risk estimates, and adverse events were gathered from patient charts. We reviewed the clinical chart in order to document the most serious index offence leading to the index hospitalization (e.g., a finding of NCRMD/unfit). If there were multiple offences, we coded the most serious offence using the Crime Severity Index (15). We categorized offences into homicide/attempted homicide/manslaughter, other violent crimes (e.g., assault, robbery, etc.; using the Uniform Crime Reporting 2 coding of violent offences against a person (16)), and all other crimes (e.g., property crimes). Purpose and characteristics of SSCOs and the prevalence and nature of adverse events were collected from rehabilitation department records. Rehabilitation records contained both quantitative data about SSCOs (e.g., documenting the number of SSCOs and attendance by date) and qualitative data (e.g., destinations, staff comments about patients’ behaviors). Adverse events were documented as present or absent for each patient during each outing. An adverse event was defined as violence toward others, suicidal behaviors or non-suicidal self-injury (described in the START as self-harm), or substance use (which were operationalized according the START definitions (17)), as well as any criminal offence, police contact during the outing, or breaching conditions (e.g., unauthorized absence from hospital). Research assistants collected the relevant data and entered it into REDCap, an electronic research data capture tool (18, 19).

### Measures

#### Short-term assessment of risk and treatability (START)

The START (17) is a structured professional judgment (SPJ) tool for the assessment of multiple adverse outcomes (i.e., violence, self-harm, suicide, unauthorized leave, substance abuse, self-neglect, and victimization) in the short-term (generally the upcoming 3 months). The START consists of 20 dynamic items (e.g., social skills, impulse control, insight) that are assessed as both strengths and vulnerabilities on a scale of 0 to 2 (0 = minimally present; 1 = moderately present; and 2 = maximally present; 17). In addition to the 20 items, assessors use additional historical information and signature risk signs in formulating risk estimates for each adverse outcome. The summary risk estimates for the seven domains are coded as low, moderate, or high. The psychometric properties of the START have been well documented in a recent review (20).

STARTs were completed by the patients’ treatment teams as part of routine clinical care and obtained from each patient’s chart. Hospital policy requires that STARTs are completed every three months for patients. START assessments are also required to be included when making an application to the P&P committee for community access for a patient. We included the START that preceded the first SSCO or the first START in the study period if the patient was already participating in SSCOs at the beginning of the year. For the START summary risk estimates, we opted to combine moderate and high risk estimates in our reporting as our goal was to describe the proportion of the sample that carried an overall elevated risk on the particular risk dimension.

### Analysis

Analyses were completed using SPSS Version 22.0 for Mac. Sociodemographic and clinical characteristics of participants were analyzed descriptively (e.g., frequencies, means, standard deviations, and ranges). SSCO purposes and staff comments identifying patient progress were analyzed qualitatively using a deductive approach (21, 22). We reviewed the comments to develop familiarity with the data and then categorized purposes of the SSCOs based on the locations attended and the staff comments indicating the activities that were participated in. We identified four primary codes relating to the purposes of SSCOs: (1) skills for daily living (e.g., grocery shopping, social skills outings), (2) vocational skills (e.g., volunteering at a food bank), (3) leisure skills (e.g., going to a library or movie theatre), and (4) health promotion (e.g., walking, hiking). We then compared our initial codes to the broader literature on psychosocial rehabilitation in psychiatric settings to ensure alignment with current best practices and research (23–25). We reviewed the staff comments again to identify notes commenting on patient performance in these areas and generated two subcodes: (1) skill development demonstrated, or (2) skill development required. Staff notes were analyzed by two co-authors independently, with a third co-author resolving discrepancies. Comments could be coded with both subcodes if improvements and challenges were noted in the same comment. Finally, adverse events were analyzed descriptively.

## Results

### Sociodemographic, clinical, and risk characteristics

A total of 110 patients took part in SSCOs in the one-year study period and were included in the sample. **Table 1** presents the characteristics of the sample. Overall, patients taking part in SSCOs were primarily male, Caucasian, and had a mean age of 41 years. In terms of prior mental health and criminal justice system contacts, the majority of patients (94.5%) had civil psychiatric involvement (e.g., inpatient admissions, emergency department visits for a psychiatric purpose) prior to the current forensic psychiatric services admission and most (75.5%) had prior criminal justice involvement (e.g., arrests, charges, or incarceration). Prior to this admission, about one-in-ten patients had a previous NCRMD or Unfit finding. Approximately, one-third of the sample had undergone a previous assessment and/or treatment in the forensic system but were not treated under a NCRMD or Unfit order (e.g., assessed for NCRMD or fitness but found not found NCRMD or unfit, respectively).

**Table 1:** Sociodemographic and clinical characteristics of the sample.

Characteristics	N (110) (%)
Gender Male Female	96 (87.3) 14 (12.7)
Age Mean (*SD*) Range	40.5 (12.8) 20 – 72
Ethnicity Caucasian Indigenous Asian Other	74 (67.3) 14 (12.7) 9 (8.2) 13 (11.8)
Education Level 8^th^ grade or less 9^th^ – 11^th^ grade High school Post-secondary/trade Unknown	13 (11.8) 40 (36.4) 24 (21.8) 32 (29.1) 1 (0.9)
Prior Contact with Services Previous NCRMD or Unfit Finding Previous Forensic Assessment or Treatment Criminal justice involvement Civil psychiatric involvement	13 (11.8) 36 (32.7) 83 (75.5) 104 (94.5)
Time under Review Board jurisdiction Mean years (*SD*) Range (years)	6.6 (8.6) 0 – 42
Primary Psychiatric Diagnosis Schizophrenia spectrum/psychotic disorders Mood disorders Neuropsychiatric disorders Other psychiatric diagnosis	91 (82.7) 7 (6.4) 8 (7.3) 4 (3.6)
Secondary Psychiatric Diagnoses Schizophrenia spectrum/psychotic disorders Mood disorders Substance use disorders Neuropsychiatric disorder Personality disorders/traits Other psychiatric diagnosis	86 (78.2) 11 (10.0) 3 (2.7) 77 (70.0) 33 (30.0) 38 (34.5) 15 (13.6)
Most Serious Index Offence Homicide/attempted Homicide/manslaughter Other violent crimes (e.g., assault, robbery, etc.) All other crimes (e.g., property crimes)	25 (22.7) 65 (59.1) 20 (18.2)
Legal Status NCRMD Unfit Involuntary	107 (97.3) 2 (1.8) 1 (0.9)
START Summary Risk Estimates: Moderate/High Violence Unauthorized Leave Suicide Self-Harm Substance Abuse Self-Neglect Victimization	82 (74.6) 55 (50.0) 15 (13.6) 20 (18.2) 73 (66.4) 74 (67.3) 56 (50.9)

NCRMD, not criminally responsible on account of mental disorder.

Most participants were diagnosed with a schizophrenia spectrum disorder (82.7%). The most common comorbid psychiatric disorder was a substance use disorder (70.0%). Over half of the sample had committed a violent offense against a person leading to their current hospitalization (59.1%) and nearly one-quarter (22.7%) had an index offence of homicide, attempted homicide, or manslaughter. The majority of participants were being treated under an NCRMD designation (95.5%). As can be seen in **Table 1**, more than half of the sample had START risk ratings for violence in the moderate or high categories (74.6%). Half of the sample (50.0%) presented with an elevated risk for unauthorized leave. The least common outcomes of concern were suicide (13.6%) and self-harm (18.2%). Many of the patients were also considered to be at moderate to high risk of substance use (66.4%), self-neglect (67.3%), and/or victimization (50.9%).

### Staff Supported Community Outing (SSCO) purpose and characteristics

A total of 463 SSCOs were conducted throughout the year with a mean of 38.6 each month (range = 31-51 SSCOs per month). Approximately one in five SSCOs (18.6%) were outings comprised of a single patient supervised by one or more staff. These were outings designed to assess the patient’s suitability for future outings and/or for patients who required multiple staff to ensure adequate and appropriate supervision; these outings were typically up to two hours in duration (see **Table 2**). Nearly a third of the SSCOs (31.3%) were small group outings, generally comprised of two to four patients who were supervised by two staff. In these instances, the outing may have required increased staff supervision (patient to staff ratios) compared to most SSCOS (e.g., given the location) or the clinical evaluation of the patients had indicated they required small group settings. These small group outings were up to four hours in duration. Finally, about half (50.1%) of the SSCOs over the one-year period consisted of outings with two staff who were responsible for up to eight patients. These large group SSCOs involved patients who were deemed suitable to be in the community with minimal supervision and in larger groups; these outings were up to six hours in duration. On average, participants went on 10.2 SSCOs per year (Median = 6, Mode = 1, SD = 10.0, range from 1-50).

**Table 2:** Description of SSCO types based on number of staff, maximum number of patients, and duration.

SSCO Type	Number of Staff	Maximum Number of Patients	Maximum Duration (hours)
Assessment	≥ 1^1^	1	2
Small	2	4	4
Large	2	8	6

^1^Assessment SSCOs may consist of one or more staff accompanying one patient based on risk factors, historical data, or other clinically relevant information.

The most frequent purpose for SSCOs included developing patients’ skills for daily living (60.4%), followed by health promotion skills (25.7%), vocational skills (13.0%), and leisure skills (7.4%). Outings to build daily living skills most commonly included grocery shopping, social skill activities such as ordering from a coffee shop, learning to take public transit, and shopping for clothes, groceries, and other personal needs. Health promotion skills included activities such as walks, bike rides, and hiking. Vocational skills focused SSCOs most commonly consisted of patients engaging in volunteer activities, for example working at a soup kitchen or a food bank. Finally, leisure skills focused SSCOs generally involved recreational activities such as bowling, golfing, going to a movie theatre or borrowing a book from the local library.

There were 544 clinical notes found in the clients’ rehabilitation files associated with the SSCOs and spoke to how a patient was functioning in terms of the four rehabilitation purposes (see **Table 3**). Most comments indicated an improvement in the individual’s skill (376 out of 544 comments, 69.1%). Staff notes often focused on skills for daily living (465 comments, 85.5% of comments). There were 317 comments highlighting improvements in patient skills, and 148 comments suggesting that there were challenges noted indicating that further opportunities were needed for skill development such as personal grooming and hygiene, specifically one note indicated a patient had extensive stains on the shirt they wore on the outing. For example, another clinical note referred to successful development of skills for daily living included the following comment: *“The patient demonstrates good skills in the area of price comparison and can identify the different sections in a grocery store and what types of products would be located in those sections”.*

**Table 3:** Frequency of SSCOs by intended purpose and staff comments.

	SSCO Purpose: Intended Skill Development *N* (%)				
	Skills for Daily Living	Vocational Skills	Health Promotion Skills	Leisure Skills	Total
Clinical notes available	465 (85.5)	13 (2.4)	32 (5.9)	34 (6.2)	544
Skill development demonstrated	317 (84.3)	13 (3.5)	16 (4.3)	30 (7.9)	376
Skill development required	148 (88.1)	0 (0.0)	16 (9.5)	4 (2.4)	168

### Adverse events

Our final objective was to examine the extent to which staff and patients on SSCOs are able to avoid adverse outcomes. There were no instances of physical violence or threats of violence toward staff, individuals in the community, other patients, or property during SSCO outings over the one-year period. Further, there were also no instances of suicidal behaviors or non-suicidal self-injury, substance use, or criminal offending.

There was one SSCO during the study timeframe which resulted in a breach of conditions and police contact. Specifically, during the SSCO, the patient absconded from staff. One week later, the patient was located by the local police and returned to hospital. There was no evidence that the patient had engaged in substance use, violence, or any other disruptive behaviors in the community, and there were no charges following the unauthorized leave. Records indicated the patient was unwilling to discuss the unauthorized leave during the SSCO, other than describing it as an impulsive decision and feeling “pressured” by clinical staff during the outing. This patient had a history of two prior unauthorized leaves, however those events had not occurred during SSCOs.

## Discussion

Forensic psychiatric patients found NCRMD are admitted to hospital recognizing that their index offences were a direct result of their mental illness and they are thus in need of treatment and rehabilitation. The provincial Review Boards must take into account the safety of the public, the mental condition of the accused, the reintegration of the accused into society, and the other needs of the accused, however, the courts recently affirmed the safety of the public is the paramount concern (26). The majority of people found NCMRD or unfit to stand trial are generally detained in custody and treated in a forensic psychiatric hospital. Community access is a slow and gradual process involving repeated assessments of the person’s risk to themselves and the community and a step-wise approach to testing the patient’s appropriateness and capacity to tolerate various environments. As we have outlined here, patients are initially tested in contexts that limit stressors for the individual, that lend themselves to staff monitoring, and that present little risk to the public. For instance, a patient’s first SSCO is likely to be to an outdoor setting such as the trails and parks near the hospital as opposed to attending an indoor setting that might be busy, noisy, and/or crowded. In addition, early SSCOs have a high staff to patient ratio (e.g., 2 to 1) to ensure that the staff are able to control the patient’s behavior and return them to hospital without incident, should the need ever arise. Mental health professionals working in forensic psychiatric systems are tasked with making treatment decisions, legal recommendations (e.g., suggestions to the Review Board regarding orders and conditions) and assessing and managing risk, and there often is a high level of uncertainty when making these decisions (27). These responsibilities can become quite complex when decisions are being made about increasing privileges and allowing for greater community reintegration, as the safety of the public becomes a more salient consideration in clinical decision making. The purpose of this study was to understand the characteristics of patients who participate in SSCOs, the purpose and rehabilitative nature of the outings, and document any adverse events that occurred during the outings over a one-year period.

On average, the patients attending SSCOs were long stay patients with severe and complex mental illness and concurrent substance use disorders who had been found NCRMD. These participants had considerable mental health and criminogenic risks and needs. Specifically, 80% of patients had committed a violent index offence, and 20% of the sample had an index offence of homicide/attempted homicide. The majority of patients had been assessed as presenting an elevated risk for violence, unauthorized leave, substance abuse, self-neglect, or victimization during the year in which they attended SSCOs. This profile of forensic psychiatric inpatients is generally consistent with the broader literature (28–30), suggesting that our findings are likely generalizable to other forensic psychiatric cohorts.

The results also suggested that there was a rehabilitative aspect to these outings, which focused on exploring various settings in the community with the goal of building skills for community re-integration. The extant literature suggests that forensic rehabilitation should foster the development of skills related to living outside of an institution (31). A large number of SSCOs focused on skills for daily living (ranging from grocery shopping, ordering a coffee at a business, to social outings), but importantly there was a variety of purposes noted, including also focusing on health promotion, vocational skills, and leisure. Many patients demonstrated an improvement in these skills, as per staff comments, suggesting that some skill development in these areas occurs on outings. Interestingly, there were a relatively low number of outings that had a leisure component, however there may have been a greater focus on health promotion with more high intensity recreational activities. Providing opportunities for exercise, socialization, and planning (time and financial management) is important given the poor educational attainment, health problems, negative symptoms and cognitive deficits seen in people with schizophrenia and specifically, a large proportion of the forensic population (32). However, it is also important to have activities that are focused on leisure and recreation (e.g., bowling, going to a library, walking in a park) as these opportunities are critical to risk mitigation and recovery; recreation/leisure is one of the central eight risk factors in the RNR model (9, 33). There were also few vocational outings across the year, however, it may be that these outings require a higher level of skill and therefore occur later in a patient’s recovery and may also be unsupervised by staff as they occur in workplace settings, which our study did not capture.

The results of this study, overall, demonstrated that supervised access to the community for a forensic sample can be done in a safe manner, while also focusing on reintegration and skill development, which supports mental health recovery. Despite the characteristics of the patient sample, and the considerable needs across the entire year, there were no episodes of violence, substance use, suicidal behaviors or criminal offending in the community. There was just one instance of a patient absconding during the outing; furthermore, there was no evidence suggesting the individual engaged in any aggression or substance use during their unauthorized leave. The low base rate of adverse events is an important finding that can impact not only policy but also inform clinicians about the frequency of adverse events during outings, which in turn, can support clinicians to make more informed decisions (34). It is crucial that these decisions are carefully balanced to prevent decisions that inappropriate restrict a patient’s rights (e.g., preventing a patient from participating in community-based outings for fear of violence despite a low base rate) or place the public at risk.

### Limitations and future directions

This study is limited due to the cross-sectional, retrospective design. Specifically, there was considerable missing information regarding patients’ behavior during outings and the extent to which the intended purposes of the outings were achieved. Given that this is a cross-sectional study, our findings are not able to inform whether these community excursions have any impact on patients’ mental health recovery. Future research could expand on this field of work, for example, by employing a prospective design which would provide the opportunity to identify multiple purposes of the SSCOs and implement measures to examine change in dynamic risks over time. Incorporating this design would also allow for exploration into the length of time between Review Board approval for community access and the patient’s first excursion into the community. Additional research could also investigate the relationship between level of engagement in community outings and post discharge outcomes (e.g., rehospitalization rates, recidivism, etc.). Second, this study focused solely on supervised access to the community for forensic psychiatric patients. Supervised outings make up one type of leave for forensic psychiatric patients, for example, patients can also be granted unescorted access to the community. Understanding the rehabilitative nature of unescorted excursions and exploring any behavioral health concerns during this type of outing can further drive policy and practice development. Finally, this study did not allow us to explore clinicians’ and patients’ perceptions of SSCOs. Future research could investigate the perceived benefits of community outings, or challenges that were associated with the decision-making process for determining patient access to the community during a period of detention. This would be valuable information, which could inform policy and practice and lead to a better understanding among treatment teams about the factors considered when making decisions for participation in community-based programming.

Future research should employ a prospective mixed methods design to address additional issues such as the factors influencing clinical decision making involved in granting access to the community from the perspective of a variety of stakeholders, including psychiatrists and other clinicians, hospital leadership, and Review Board members. It would be particularly helpful to understand the ways in which these individuals are influenced by risk, rehabilitation, medicolegal concerns, and even media and policy makers when making their decisions.

## Conclusion

It is important for mental health clinicians and Review Boards making decisions about access to the community for forensic psychiatric patients to make evidence-based risk and rehabilitative decisions. These decisions can be challenging given the need to prioritize the safety of the public and yet consider the therapeutic benefits provided by community outings, individual liberties of the patient, and the high level of media attention when an adverse event occurs in the community. We found that SSCOs had clear treatment objectives that correspond to existing theory (RNR, (9)) and risk items on established measures (e.g., START, (17)). Overall, this study demonstrates that despite their complexity, the forensic psychiatric patient population, with considerable risk characteristics, can safely participate in SSCOs with careful risk mitigation strategies that prioritize public safety and provide essential patient skill development and mental health recovery.

## Data availability statement

The raw data supporting the conclusions of this article will be made available by the authors, without undue reservation.

## Ethics statement

The studies involving humans were approved by University of British Columbia. The studies were conducted in accordance with the local legislation and institutional requirements. The ethics committee/institutional review board waived the requirement of written informed consent for participation from the participants or the participants’ legal guardians/next of kin because this study was a retrospective chart review of clinical files, considered minimal risk by ethics board. No interviews of human participants.

## Author contributions

CF: Data curation, Formal analysis, Investigation, Methodology, Writing – original draft, Writing – review & editing. KP: Data curation, Formal analysis, Investigation, Methodology, Project administration, Supervision, Validation, Writing – original draft, Writing – review & editing. PH: Conceptualization, Investigation, Validation, Writing – original draft, Writing – review & editing. TN: Conceptualization, Data curation, Investigation, Methodology, Supervision, Validation, Writing – original draft, Writing – review & editing.
